# Supplementary material for: Assessing the Integrative Framework for the Implementation of Change in Nursing Practice: Comparative Case Studies in French Hospitals
Source: Healthcare (Basel). 2022 Feb 23;10(3):417. doi: 10.3390/healthcare10030417 (PMC8953539; doi:10.3390/healthcare10030417)
Supplement: Supplementary file 1 [file healthcare-10-00417-s001.zip › healthcare-1582374-supplementary.pdf]

Assessing the Integrative Framework for the Implementation of Change in Nursing Practice: Comparative Case Studies in French Hospitals

Supplementary Material

Table S1: Study reporting using COREQ checklist. To report this study, we used the "Consolidated criteria for reporting qualitative research" COREQ checklist (1), we added a new column to the 32 item checklist and in which we answered about our study, the guide questions which described each item from the checklist.

No	Item	Guide Questions/Description	Study Reporting
**Domain 1: Research team and reflexivity**			
**Personal Characteristics**			
**1.**	Interviewer/facilitator	Which author/s conducted the interview or focus group?	The corresponding author
**2.**	Credentials	What were the researcher's credentials? *E.g. PhD, MD*	Registered Nurse "RN" and PhD student
**3.**	Occupation	What was their occupation at the time of the study?	forth year PhD student
**4.**	Gender	Was the researcher male or female?	Female
**5.**	Experience and training	What experience or training did the researcher have?	Experience in nursing sciences, healthcare system and quality management. Training on study design and methodology qualitative and quantitative data analysis.
**Relationship with participants**			
**6.**	Relationship established	Was a relationship established prior to study commencement?	There is no relationship established prior to study
**7.**	Participant knowledge of the interviewer	What did the participants know about the researcher? e.*g. personal goals, reasons for doing the research*	Participants only know a general intention of the authors in terms of the study aim and objectives: which is understand the implementation process of certification procedures in the hospital. .
**8.**	Interviewer characteristics	What characteristics were reported about the interviewer/facilitator? e.g. *Bias, assumptions, reasons and interests in the research topic*	The interviewees were carried only by the principle investigator (PI). Nurses' interviews were conducted in their working shift, alone and in private space.
**Domain 2: study design**			
**Theoretical framework**			
**9.**	Methodological orientation and Theory	What methodological orientation was stated to underpin the study? *e.g. grounded theory, discourse analysis, ethnography, phenomenology, content analysis*	Study used deductive approach<br>The methodological orientation was a content analysis of discourses using the Integrative framework for implementing change into nursing practice IFINP

**Participant selection**			
**10.**	Sampling	How were participants selected? *e.g. purposive, convenience, consecutive, snowball*	Participants were selected in a purposive way, in the first step they have to be involved in the process of implementation of certification, in the second step only nurses were interviewed because we interested to study the nurse role are engaged in the process of implementation in their daily workflow. All interviews were carried after confirmation to participate in the study
**11.**	Method of approach	How were participants approached? e*.g. face-to-face, telephone, mail, email*	Face to face semi structured interviews,
**12.**	Sample size	How many participants were in the study?	Thirty- three
**13.**	Non-participation	How many people refused to participate or dropped out? Reasons?	No one
**Setting**			
**14.**	Setting of data collection	Where was the data collected? e*.g. home, clinic, workplace*	In the workplace at hospital
**15.**	Presence of non-participants	Was anyone else present besides the participants and researchers?	Participants were interviewed alone and in a private place generally
**16.**	Description of sample	What are the important characteristics of the sample? *e.g. demographic data, date*	For nurses the average of experience is 9 years in theirs services and 43 years old. All have same qualification level of studies “RN”
**Data collection**			
**17.**	Interview guide	Were questions, prompts, guides provided by the authors? Was it pilot tested?	Interviews were conducted according to a semi structured interview guide. Tested on nurses out of the studied hospital
**18.**	Repeat interviews	Were repeat interviews carried out? If yes, how many?	No
**19.**	Audio/visual recording	Did the research use audio or visual recording to collect the data?	Yes, all interviews were recorded
**20.**	Field notes	Were field notes made during and/or after the interview or focus group?	After each interview, a brief notes were registered for the important emergent ideas
**21.**	Duration	What was the duration of the interviews or focus group?	Leaders and managers: 30-45 min<br>Nurses: 20-30
**22.**	Data saturation	Was data saturation discussed?	Yes, interviews have been stopped, when their output reach a closing sense
**23.**	Transcripts returned	Were transcripts returned to participants for comment and/or correction?	No
**Domain 3: analysis and findings**			
**Data analysis**			
**24.**	Number of data coders	How many data coders coded the data?	coding data were used in limited part of the study,<br>and was conducted manually by the corresponding author and revised by the co-author

**25.**	Description of the coding tree	Did authors provide a description of the coding tree?	Yes the used code were described briefly in legend of table
**26.**	Derivation of themes	Were themes identified in advance or derived from the data?	themes were derived from the analysis of interviews
**27.**	Software	What software, if applicable, was used to manage the data?	Data were analysed manually on excel sheets
**28.**	Participant checking	Did participants provide feedback on the findings?	No
**Reporting**			
**29.**	Quotations presented	Were participant quotations presented to illustrate the themes / findings? Was each quotation identified? e.*g. participant number*	Yes, some of the participants quotations are presented in the manuscript to support the themes and findings, and it identified according to the professional respondent according to a defined acronym and a sequence number
**30.**	Data and findings consistent	Was there consistency between the data presented and the findings?	Yes there was a consistency between the emergent themes, the used tools and the findings are translated in an implementation framework
**31.**	Clarity of major themes	Were major themes clearly presented in the findings?	In the discussion part of the study while presenting the developed framework
**32.**	Clarity of minor themes	Is there a description of diverse cases or discussion of minor themes?	Not applicable

1. Tong, A.; Sainsbury, P.; Craig, J. Consolidated criteria for reporting qualitative research (COREQ): A 32-item checklist for interviews and focus groups. *Int. J. Qual. Health Care* **2007**, *19*, 349–357.
